# Supplementary material for: Chromothripsis during telomere crisis is independent of NHEJ, and consistent with a replicative origin
Source: Genome Res. 2019 May;29(5):737–49. doi: 10.1101/gr.240705.118 (PMC6499312; doi:10.1101/gr.240705.118)
Supplement: Supplemental Material [file supp_gr.240705.118_Supplemental_file_1.zip › contigs/annotated_contigs/DB51/contig.4.DB51_length_955_mean_cov_5.60523560209.docx]

**DB51_length_955_mean_cov_5.60523560209**

```
ATCACATCAAGTTAAAAAAGCTTCTGCACTGAAAAAGAAACAATCAACAAAGTGAAGGAACAACCCACAGAATGGTAGAAAATATTTGC
 >chr6:145789924-145790254 + E=1e-181 p=2e-02
TAACTATCCATCTGACAAGAGATTAATAACCAGAATACATAAGGAGCTCAAACAACTATATAGAAAAATAACTAATAATCTGATTTTAA

AATGGGCAAAAGATCTTAATAGACATTTGTGTTAGTCCATTTTCATACAGCTATAAAGAACTGCCTGAGACTGAGTAATTTATAAAAGA

AAGAGGTTTAATTGACTGACAGTTTCACATGGCTGGAGAAGCCTCAGGAAACTTACAATT|AT|ATAAAAATATGCTCAACATCATTGA
                                                             >chr13:109305481-109305509
T|GCCAATGATAATT|GGCAAGTACTGACCTTCTGGCTGGCTGTCACAGTGAGAATCTTTTCTGGCATTGGTGGCCCAACATTTTAACT
 - E=6e-02     >chr20:20144871-20145362 + E=2e-280
TTACAGAGCCAGTAATTTCCAAACCTGGCCCACATCCTAATCTCCTGGGGAATTTTTTAAAACACAAATTCCTAAGCCCCATCCAGAGA

TTCTAGTCCATCAAATTTTAACAAGGCCTACAGAAGTGGGCTAGAAATCTGGCCTACCTATGTAACAATTGGGAATCTGGAAAATTTTC

ACTTACATAACCACATGCTGCTCCCATTCTGTAAAGTTTGCATCTCTTGGATAATTTGAATTTGGGTTACTGGTTGTCTAGAGTCTTTG

GTTGCAAGTAACAGGAAATCCAACCAAACTGGCTTTGAAAAAAAAATTGAATAATTGCCTCACATAACTAAGGATAACTCTGGTGGTGA

GACTGTTTTAAATATGGCTTGATCTAGGATTTATTTAGATTACCAAATCAAGGACCAA|GTTT|TAATGTTTAGTTCCCACAAATACAT
                                                           >chr6:145791009-145791109 -
GAGAACATGCAAAGTTTCTCTTTCTGTGCCTGGCTTTATCACTTAACATAATGATCTCAAGTTCCATCCATE=3e-48
```
